# A Multicenter, Randomized, Double-Blind, Placebo-Controlled Study of the Effects of Loki zupa in Patients With Chronic Asthma

**DOI:** 10.3389/fphar.2018.00351

**Published:** 2018-04-10

**Authors:** Yubao Lv, Ying Wei, Muhammadjan Abduwaki, Tohti Jurat, Fengsen Li, Huaizhen Wang, Yuhua Wu, Zheng Li, Bo Liu, Hongjun Yin, Yuxue Cao, Mammat Nurahmat, Zihui Tang, Jingcheng Dong

**Affiliations:** ^1^Department of Integrative Medicine, Huashan Hospital, Fudan University, Shanghai, China; ^2^Institutes of Integrative Medicine, Fudan University, Shanghai, China; ^3^Xinjiang Uygur Medical College, Hotan, China; ^4^The Traditional Chinese Medicine Hospital Affiliated to Xinjiang Medical University, Ürümqi, China; ^5^Department of Respiratory Medicine, First People’s Hospital of Kashi, Kashi, China; ^6^Department of Respiratory Medicine, Second People’s Hospital of Kashi, Kashi, China; ^7^Department of Respiratory Medicine, Xinjiang Production and Construction Corps Seventh Division Hospital, Kuytun, China

**Keywords:** Loki zupa, asthma, double-blind, clinical trial, placebo-controlled

## Abstract

The purpose of this study was to evaluate the efficacy and safety of Uyghur medical formula Loki zupa in patients with chronic asthma. Adult patients with chronic asthma randomly received placebo or Loki zupa as add-on to inhaled corticosteroids (ICS) maintenance treatment. Loki zupa or mimics was administered orally 10 ml per time, three times a day for 8 weeks. The primary endpoints were asthma control test (ACT) score and peak expiratory flow (PEF). The secondary endpoints were acute exacerbation rate, lung function, night waking days, and symptom-free days in the near 2 weeks, Asthma Quality of Life Questionnaire (AQLQ) score and some inflammatory cytokines in peripheral blood. A total of 240 adult patients with chronic asthma were enrolled, and 218 patients were randomized to placebo (*n* = 109) or Loki zupa (*n* = 109) in addition to ICS for 8 weeks. Treatment with Loki zupa resulted in significant improvement in ACT score compared to the placebo group (*p* = 0.002). Furthermore, oral taken of Loki zupa increased the PEF obviously (*p* = 0.026). Loki zupa treatment did not improve the forced expiratory volume in 1 s (FEV_1_, *p* = 0.131) and FEV_1_/FVC compared to the placebo treatment (*p* = 0.805). The placebo group had higher rates of acute exacerbations than the Loki zupa group (6.3% vs. 0, *p* = 0.027). Subjects randomized to Loki zupa had increased daytime symptom-free days within 2 weeks than placebo (*p* = 0.016). However, Loki zupa had no effect on night waking days in the near 2 weeks (*p* = 0.369) and AQLQ score (*p* = 0.113). No significant effect was found on inflammatory cytokines (IL-2, IL-4, IL-5, IL-10, IL-13, IL-17, IL-33, IFN-γ, and TGF-β) between the two groups (*p* > 0.05). No adverse events and severe asthma exacerbations were recorded in the two groups (*p* > 0.05). Loki zupa add-on to standard ICS produced clinically significant improvements in ACT score, PEF, daytime symptom-free days and acute exacerbation in patients with chronic asthma.

**Clinical trial:** This study is registered at http://www.chictr.org.cn/ with identifier number ChiCTR-IPR-16008106.

## Introduction

Asthma is a common, chronic respiratory disease affecting 1–18% of the population in different countries. Asthma is characterized by variable symptoms of wheeze, shortness of breath, chest tightness and/or cough, and by variable expiratory airflow limitation (Global Strategy for Asthma Management and Prevention, 2016). Inhaled corticosteroids (ICS) and β_2_-adrenoceptor agonists treatments are significantly effective in relieving asthma (Nagai, 2012; Holgate, 2013). However, current asthma therapy could not completely control the asthma symptoms and future risks, and there was still a proportion of people could not be well treated with current drugs (Partridge et al., 2011). Furthermore, some people may have exacerbations of asthma, which may be life-threatening and carry a significant burden to patients and society. There is still a number of people using asthma medications having side effects, such as oral thrush and dysphonia, and long-term and/or high-dose ICS may induce systemic side effects including easy bruising, an increase beyond the usual age-related risk of osteoporosis, cataracts and glaucoma, and adrenal suppression (Global Strategy for Asthma Management and Prevention, 2016).

Asthma is a complex and heterogeneous disease, with different underlying disease processes. Traditional Chinese medicine is considered to have the potential to regulate complex disease processes through multiple targets and pathways, and is widely used for asthma treatment in China and many other places throughout the world (Luo et al., 2014; Wang et al., 2014; Wei et al., 2015). As one kind of traditional Chinese medicine, Uyghur medicine is frequently used by Uyghur people to treat diseases in the northwest of China. Loki zupa, which consists of the roots of *Hyssopus cuspidatus* Boriss (Shenxiangcao), Iris halophila Pall (Yuanweigen), and honey, is commonly used in Uyghur medicine for asthma. Previous investigations demonstrated that Loki zupa was the best and strongest formula to treatment respiratory diseases in Uyghur traditional medicine. Animal research results showed that Loki zupa decoction reduced airway hyperresponsiveness, attenuated airway inflammation, promoted Th1, and suppressed Th2 cell functions in an OVA-induced asthma mouse model (Wei et al., 2016). However, the clinical evidences of the efficacy of Loki zupa in asthma patients are rare. In this study, we evaluated the effects of Loki zupa in patients with chronic asthma.

## Materials and Methods

### Design and Eligibility

A multicenter, randomized, double-blind, placebo-controlled study as add-on to ICS maintenance treatment was performed. Asthma was diagnosed according to Global Initiative for asthma (GINA) 2014 and the Chinese guidelines published in 2008 for the diagnosis and management of asthma. Eligible subjects were all genders between the ages of 18 and 70 years, asthma control test (ACT) score lower than 20 and continuous use of ICS (or has an supplement of ICS) for more than 3 months. To be included in this trial, the subjects had to complete the asthma diary records in the introduction stage. Besides, the subjects were required not to have upper or lower respiratory tract infection within 2 weeks and not oral or intravenous use of glucocorticoid therapy. All subjects agreed to the study protocol and signed a written informed consent. According to the informed consent sheet, all participations were full voluntary and they received adequate information which included that they were free to withdraw from the trial at any time. The main exclusion criteria were subjects with severe duration and acute episodes that need oral use of glucocorticoid. Subjects combined with COPD, pulmonary fibrosis, bronchiectasis, active tuberculosis, pleural effusion, pulmonary embolism, and history of pneumonectomy were also excluded. Subjects were excluded if they were pregnant or were planning to become pregnant as well as breast feeding, were malignant tumor or hematopathy patients, participated in other drug trials, heart, liver, and kidney damage seriously (cardiac function grade 3 ∼ 4, ALT and/or AST above 1.5 times more than normal ceiling, above upper limit of normal). Subjects with conditions that were not proper to be included were also excluded by researchers. The study protocol was approved by the appropriate independent ethics committees. The trial was registered at http://www.chictr.org.cn/ with identifier number ChiCTR-IPR-16008106.

### Randomization

**FIGURE 1:**
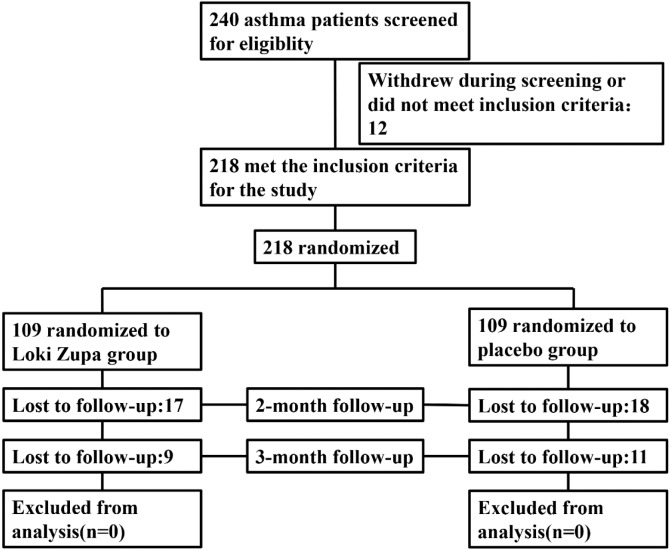
Study flow chart.

Eligible patients were randomized (in a 1:1 ratio) to either the Loki zupa treated group or the placebo-controlled group (**Figure 1**). The randomization sequence was computer-generated and the study medication was labeled with sequential randomization numbers. The investigators, study subjects, and study site personnel were all blinded to treatment allocation. Loki zupa or mimics were supplied in different kits labeled with unique code numbers. Randomization codes were concealed from all of the staff members and participants at the investigational sites.

### Study Procedure

Subjects were enrolled between July 2014 and August 2015 from outpatient in five hospitals in China. After a 2-week screening period, eligible patients were randomly divided into Loki zupa treated group and the placebo-controlled group. Three visits were included in the whole protocol, including a visit at the start of the trial (screening), a visit at randomization (baseline), and a visit at the end of the 8-week treatment. On the basis of ICS, Loki zupa treated group received Loki zupa, and the placebo-controlled group received Loki zupa mimics. Loki zupa or mimics were administered orally 10 ml per time, three times a day for 8 weeks.

### Preparation of Loki zupa and Mimics

**FIGURE 2:**
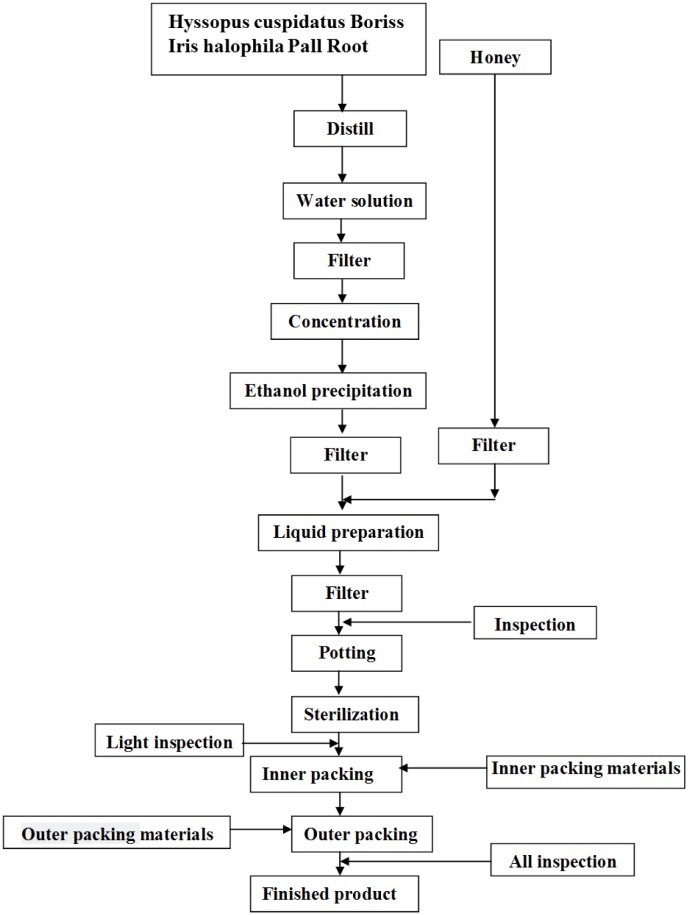
Flow chart of Loki zupa preparation process.

Loki zupa or mimics were produced by Xinjiang Uygur Medical College affiliated pharmaceutical factory, and were approved by State Food and Drug Administration (SFDA) according to the quality standards of SFDA. Preparation of Loki zupa was referred to the literatures (Encyclopedia of medical science in China, 2009; Aitaer, 2010). As shown in **Figure 2**, herbs of Hyssopus cuspidatus Boriss (300 g) and Iris halophila Pall Root (150 g) were decocted in water for 1.5 h twice, and 300 ml distillate was collected for further usage. The decoction was merged, filtered, and concentrated. The concentrated decoction was combined with the collected distillate, followed by the addition of 100 ml honey. Distilled water was added to adjust the amount to 1000 ml. The above solution was then mixed, filtered, sterilized, and subpackaged for use (Product Specification 10 ml/bottle, 6 bottles/box). The placebo was prepared by diluting 100 ml honey with distilled water to 1000 ml. The caramel color was added to adjust the color of placebo to be the same as Loki zupa.

### Outcome Evaluation

Assessments were performed at the first visit and the last visit by evaluators blinded to the treatments. The primary outcomes were ACT score and peak expiratory flow (PEF). The secondary endpoints were acute exacerbation rate, lung function, night waking days, and daytime symptom-free days in the near 2 weeks, Asthma Quality of Life Questionnaire (AQLQ) score and the inflammatory cytokine levels in peripheral blood. ACT score was evaluated by the study site personnel at every visit. PEF was assessed by data from a PEF meter which could be performed by patients at home every morning. Lung function were measured by spirometry which includes forced expiratory in one second (FEV_1_) and FEV_1_/forced vital capacity (FVC) and were performed in the pulmonary function test room in each center. Night waking days and daytime symptom-free days in the near 2 weeks were investigated according to the asthma diary recorded by patients. Asthma exacerbations rate was assessed by self-reported initiation of unexpected asthma control drugs during the 8 weeks treatment and 12 weeks follow up after enrollment. AQLQ is a 32-item survey in four domains including activity limitations, asthma symptoms, emotional function, and environmental exposure. AQLQ is always used to assess the degree to which important activities have been limited by asthma during the last 2 weeks. AQLQ score is positively correlated with quality of life. Inflammatory cytokines in serum were measured by ELISA according to the manufacturers’ instructions.

### Safety

Adverse events, severe asthma exacerbations (deterioration of asthma requiring short-acting β2 agonists more than 10 spray/day for 2 days or systemic corticosteroid use or an in-patient hospitalization/emergency department visit or a reduction in FEV_1_ more than 20% of baseline) were assessed. Besides, vital signs, clinical chemistry parameters, hematology parameters, and ECGs were also evaluated for safety assessment.

### Statistical Analysis

All statistical analyses were performed with SAS software and data were analyzed according to the full analysis set principle. Wilcoxon rank sum tests were used for variables with an ordinal distribution (median and range). Continuous variables are presented as the Mean ± SD. ANOVA was used for normally distributed continuous outcomes and logistic regression was used for binary outcomes. ACT score was analyzed using Analysis of Covariance (ANCOVA) model. The numbers (%) of patients were used for description of the classification variables and the means (SD) and minimums and maximums were used for description of the quantitative variables. Values of *p* < 0.05 were considered statistically significant.

## Results

### Enrollment and Subjects Characteristics

Of 240 adult asthma patients screened, we enrolled 218 subjects who underwent randomization at five sites in China from July 2014 to August 2015. A total of 109 subjects were randomized to placebo group, and 109 subjects were randomized to Loki zupa treatment group. Baseline characteristics were similar in the two groups and shown in **Table 1**.

**Table 1:** Baseline characteristics.

Characteristic	Loki zupa (*n* = 83)	Placebo (*n* = 80)	*P*-value
Age, y	47.95 ± 11.58	50.84 ± 13.14	0.568
Male	31 (37.3%)	32 (40%)	0.728
Female	52 (62.7%)	48 (60%)	0.728
Height, cm	162.28 ± 8.47	162.64 ± 8.92	0.365
Weight, kg	69.05 ± 12.86	70.33 ± 12.80	0.087
Asthma duration	9.61 ± 10.04	7.96 ± 6.98	0.800
Asthma onset age	38.98 ± 13.69	43.18 ± 11.972	0.426
Asthma family history	16 (19.3%)	12 (15%)	0.469
Eczema family history	1 (1.2%)	2 (2.5%)	0.539
Rhinitis family history	15 (18.1%)	9 (11.3%)	0.219
Smoking history	71 (84.52%)	65 (78.31%)	0.192
Major life events	4 (4.82%)	10 (12.5%)	0.560
Heart rate	82.24 ± 8.73	80.79 ± 10.12	0.764
Body temperature	36.54 ± 0.34	36.54 ± 0.32	0.312
Respiratory rate	18.16 ± 2.88	18.10 ± 3.29	0.370
Systolic pressure	117.82 ± 11.56	118.64 ± 9.78	0.770
Diastolic pressure	75.24 ± 8.36	75.38 ± 8.57	0.974
Allergic history	18 (21.7%)	27 (33.8%)	0.085
Abnormal physical examination	13 (3.6%)	4 (5%)	0.663
ACT score	15.46 ± 0.29	15.54 ± 0.32	0.855
FEV_1_	72.959 ± 24.538	72.335 ± 29.376	0.882
FEV_1_/FVC	73.429 ± 19.885	73.488 ± 15.877	0.971
PEF	336.474 ± 149.036	348.444 ± 146.578	0.594
Symptom-free days	3.54 ± 4.457	3.71 ± 4.433	0.807
Night waking days	5.4460 ± 4.6960	5.8500 ± 4.8390	0.369
AQLQ score	112.23 ± 36.485	109.46 ± 33.92	0.617
IL-2	4.65 ± 4.27	4.64 ± 9.93	0.980
IL-4	10.08 ± 11.72	9.51 ± 11.21	0.749
IL-5	211.07 ± 141.08	223.27 ± 184.47	0.637
IL-10	13.06 ± 15.90	10.88 ± 12.91	0.336
IL-13	53.80 ± 97.01	36.29 ± 92.73	0.240
IL-17	9.58 ± 15.38	7.49 ± 11.64	0.327
IL-33	114.95 ± 111.17	100.94 ± 106.58	0.412
IFN-γ	28.87 ± 50.53	17.94 ± 35.66	0.112
TGF-β	730.33 ± 301.60	718.79 ± 315.44	0.812

*Plus-minus values are mean ± SD. n(%) represents number and percentage of the total in each group.*

### Efficacy

**FIGURE 3:**
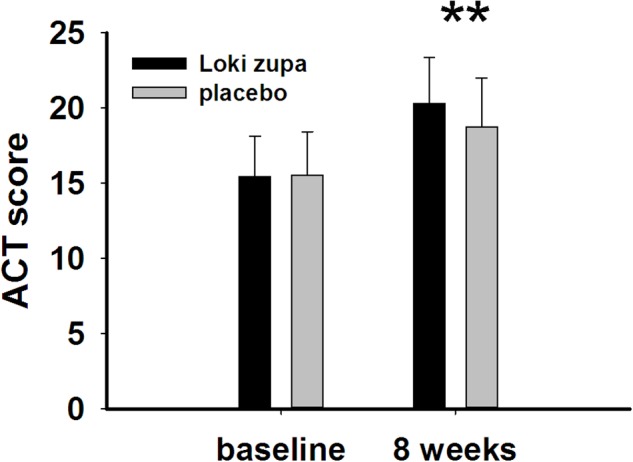
Asthma control test (ACT) score change from baseline (*p* = 0.002). ^∗∗^*p* < 0.01 vs. placebo group.

**FIGURE 4:**
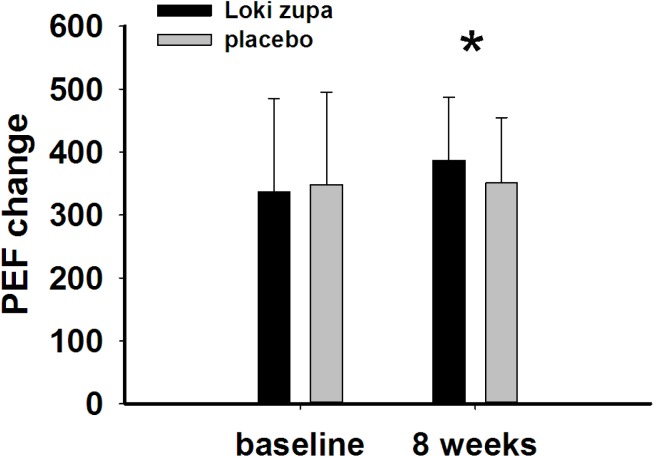
Peak expiratory flow (PEF) change from baseline (*p* = 0.026). ^∗^*p* < 0.05 vs. placebo group.

**FIGURE 5:**
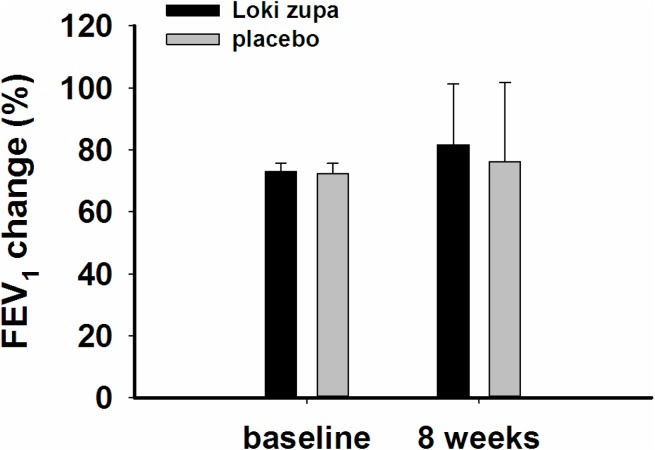
FEV_1_ change from baseline (*p* = 0.131).

**FIGURE 6:**
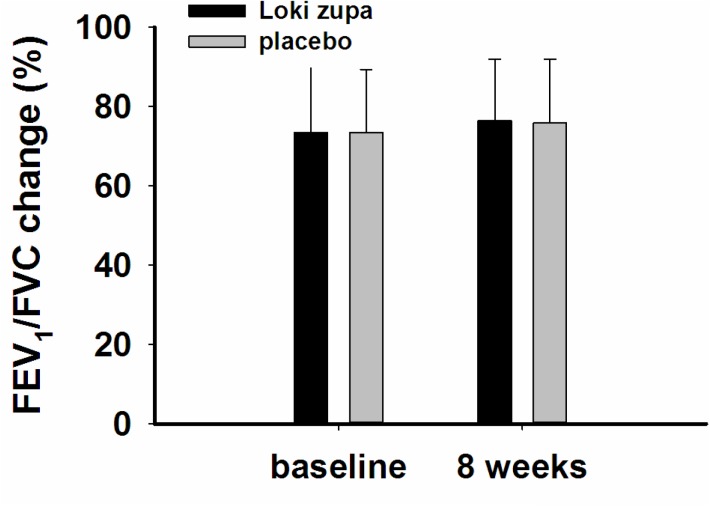
FEV_1_/FVC change from baseline (*p* = 0.805).

**FIGURE 7:**
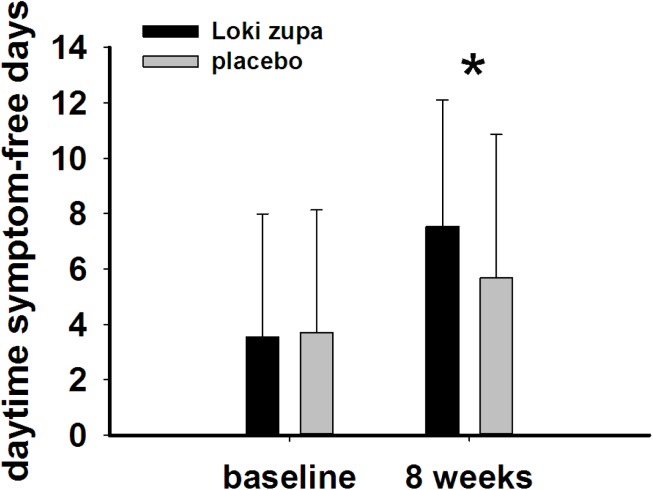
Daytime symptom-free days change from baseline in the near 2 weeks (*p* = 0.016). ^∗^*p* < 0.05 vs. placebo group.

**FIGURE 8:**
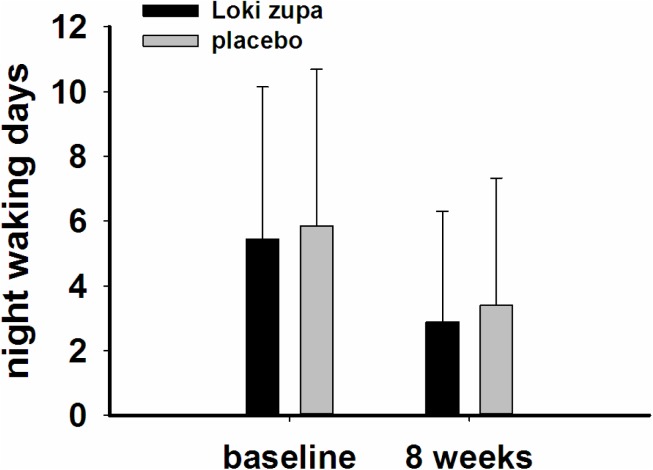
Night waking days change from baseline in the near 2 weeks (*p* = 0.369).

**FIGURE 9:**
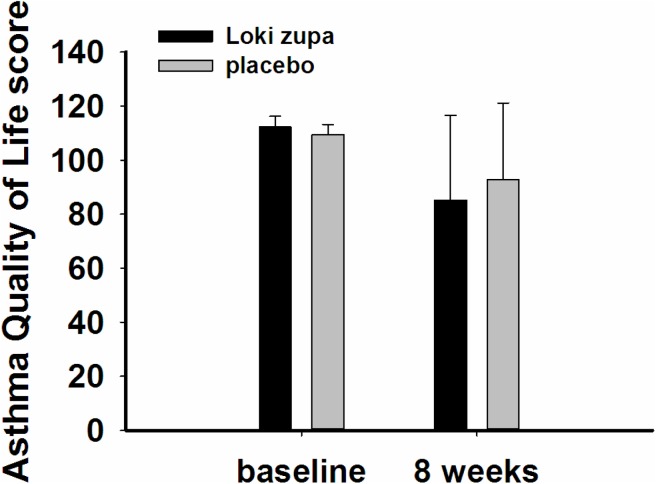
Asthma Quality of Life (AQOL) change from baseline (*p* = 0.113).

The results showed that subjects with chronic asthma randomized to Loki zupa treatment group had significant improvement in ACT score than the placebo group after 8 weeks treatment (*p* = 0.002, **Figure 3**). Furthermore, oral taken of Loki zupa caused remarkable changes in PEF compared to patients in the placebo group (*p* = 0.026, **Figure 4**). Loki zupa administration induced improvement in the FEV_1_ compared to its mimics, however, the difference was not significant (*p* = 0.131, **Figure 5**). Loki zupa did not induce improvement in the FEV_1_/FVC (*p* = 0.805, **Figure 6**) after 8 weeks treatment. Acute exacerbation rate is one of the secondary endpoints of our trial. Our results demonstrated that the Loki zupa group had lower rates of acute exacerbations than the placebo group (0 vs. 6.3%, *p* = 0.027, **Table 2**). There was significant difference in the daytime symptom-free days between the two groups (*p* = 0.016, **Figure 7**). The night waking days (*p* = 0.369, **Figure 8**) and the AQLQ score (*p* = 0.113, **Figure 9**) did not change obviously between the two groups. No significant effect was found on the inflammatory cytokines including the IL-2, IL-4, IL-5, IL-10, IL-13, IL-17, IL-33, IFN-γ, and TGF-β between the two groups (**Table 3**, *p* > 0.05).

**Table 2:** The acute exacerbations.

	Loki zupa	Placebo	Statistic	*P*-value
*N*(missing)	83 (0)	80 (0)		
Exacerbation	0 (0)	5 (6.30%)	Pearson chi-square(5.352)	0.021
No exacerbation	83 (100%)	75 (93.70%)		

*n(%) represents number and percentage of the total in each group.*

**Table 3:** The inflammatory cytokines in the serum.

cytokine	Loki zupa (*n* = 83)	Placebo (*n* = 80)	*P*-value
IL-2	4.87 @ 10.37	6.88 @ 24.68	0.508
IL-4	8.46 @ 23.11	11.33 @ 34.74	0.538
IL-5	189.37 @ 136.69	180.15 @ 138.33	0.670
IL-10	9.22 @ 10.68	9.88 @ 19.29	0.790
IL-13	29.08 @ 59.43	35.95 @ 86.49	0.558
IL-17	4.81 @ 10.02	6.68 @ 19.84	0.454
IL-33	99.57 @ 196.78	107.25 @ 280.00	0.841
IFN-γ	31.95 @ 129.57	18.55 @ 57.02	0.392
TGF-β	553.09 @ 272.14	581.06 @ 270.82	0.513

*Data were expressed as mean ± SD.*

### Safety

No adverse events and severe asthma exacerbations were recorded in the two groups. Vital signs, clinical chemistry parameters, hematology parameters, and ECGs did not change significantly from baseline between Loki zupa and the placebo groups.

## Discussion

Asthma is a common chronic respiratory disease worldwide (Harkness et al., 2015). Despite currently available therapies, many patients remain uncontrolled and asthma exacerbations may occur among a population of controlled asthmatics. Asthma control includes symptom control and future risk of adverse outcomes. Poor symptom control is burdensome to patients and is a risk factor for future exacerbations. In consideration of these aspects, we chose ACT score and PEF as our primary outcomes. Our results showed that patients randomized to Loki zupa for 8 weeks had significant improvement in ACT score than Loki zupa mimics, indicating that Loki zupa is effective in improving the symptoms of asthma and is beneficial to control asthma. Asthma is characterized by variable expiratory airflow limitation. PEF can easily be performed at home and is often used as an indicator of lung function in asthma (Pbert et al., 2012). We also chose PEF as another primary outcome to assess the response to treatment. There was significant increase in the PEF in patients receiving Loki zupa treatment, indicating the improvement in the airway obstruction. To our knowledge, our study is the first to show a beneficial association between Loki zupa administration and asthma symptom control in chronic adult asthma patients.

According to GINA report, lung function is most useful as an indicator of further risk after diagnosis of asthma (Global Strategy for Asthma Management and Prevention, 2016). Lung function was selected as one of the secondary outcomes based on the GINA report. There were no significant differences in FEV_1_ and FEV_1_/FVC between Loki zupa and its mimics. Lung function, particularly forced FEV_1_ as a percentage of predicted, is an important part of the assessment of future risk (Global Strategy for Asthma Management and Prevention, 2016). From this point of view, our results of the lung function may suggest the lack of efficacy in lowering the future risk. However, treatment with Loki zupa resulted in reduced acute exacerbation of asthma compared to the placebo, which in part may indicate the capability of Loki zupa in inhibiting the future risk of exacerbation. Besides, the lack of significant difference of lung function by Loki zupa administration may be due to the viewpoint that FEV_1_ is more sensitive and specified for larger rather smaller airways (van Dalen et al., 2008; Yamaguchi et al., 2009). Our results demonstrated a tendency in improving FEV_1_ by Loki zupa, given full consideration of the above factors, maybe future investigation with more participants and longer treatment time is needed to further clarify the efficacy of Loki zupa on FEV_1_. We found that Loki zupa was effective in increasing the daytime symptom-free days in the near 2 weeks compared to placebo, indicating the symptom control efficacy by Loki zupa. However, Loki zupa administration induced no significant differences in the night waking days and AQLQ score compared to placebo.

Asthma is a disease characterized by chronic airway inflammation (Reiter et al., 2013; George and Brightling, 2016; Shimoda et al., 2016). No significant effect was found on the inflammatory cytokines including the IL-2, IL-4, IL-5, IL-10, IL-13, IL-17, IL-33, IFN-γ, and TGF-β between the two groups. The Th2 cytokines IL-4, IL-5, and IL-13 are believed to be associated with eosinophilic inflammation (Hinks et al., 2015). Animal study had shown an anti-inflammatory effect in reducing Th2 cytokines of Loki zupa in OVA-induced asthma model challenged with OVA for 1 week (Wei et al., 2016). The discrepancy between the results of this study and the animal experiment data may indicate that Loki zupa may be effective in alleviating Th2 type inflammation with a short asthma history. IL-17 is another inflammatory cytokine secreted by Th17 cells which has been shown to contribute to the asthma disease pathology (Nakajima and Hirose, 2010; Wang et al., 2011; Zhang et al., 2015). Interleukin 33 has been identified as a trigger of Th2 cell differentiation and engaged in asthma progress (Makrinioti et al., 2014; Oczypok et al., 2015). The Th1 cytokine IFN-γ also participates in inflammation in asthma pathogenesis. Our results that Loki zupa was not capable of regulating the inflammatory cytokines suggested that the regulatory feature of Loki zupa may not be reflected in suppression of inflammation. There may be several reasons that explain the ineffectiveness of Loki zupa in attenuation of these inflammatory cytokines. First of all, the enrolled subjects were all in the chronic remission stage of asthma, and the airway inflammation is under control to some extent which could be manifested in the levels of some of the inflammatory cytokines detected from blood samples collected before treatment. Secondly, during the asthma progression, the inflammatory cytokines in the serum may be in a dynamic variation process along with the severity of inflammation. In consideration of these factors, the time of blood collection may be also influence the inflammatory cytokine levels. Thirdly, the regulatory mode of Loki zupa may not be associated with inhibition of inflammation.

The present findings suggested that Loki zupa had uniquely effective features which manifested in improvement in ACT score and PEF, increase in the daytime symptom-free days, and reduction in acute exacerbation. These in all indicated a promising intervention to treat chronic adult asthma in combination with ICS.

## Author Contributions

JD, YL, and YiW conceived and designed the study. YL, YiW, TJ, FL, HW, YuW, ZL, BL, HY, MA, YC, and MN performed the study. ZT analyzed the data. YiW wrote the manuscript. JD and YL revised the manuscript. All the authors read and approved the final manuscript and agreed to be accountable for all aspects of the work.

## Conflict of Interest Statement

The authors declare that theresearch was conducted in the absence of any commercial or financial relationships that could be construed as a potential conflict of interest.
